# Hospital challenges and managerial approaches to combat COVID-19 outbreak: a qualitative study in southeastern Iran

**DOI:** 10.1186/s12913-023-09631-0

**Published:** 2023-06-26

**Authors:** Zahra Ebrahimi Rigi, Zakieh Namjoo, Maryam Jabarpour, Mehdi Ahmadinejad, Maryam Ahmadipour, Parvin Mangolian Shahrbabaki, Mahlagha Dehghan

**Affiliations:** 1 Department of Nursing, School of Nursing and Midwifery, Iranshahr University of Medical Sciences, Iranshahr, Iran; 2 Shahid Bahonar Hospital, Kerman University of Medical Sciences, Kerman, Iran; 3 Clinical research unit, Shahid Bahonar Academic Center, Kerman University of Medical Sciences, Kerman, Iran; 4 Department of Anesthesiology, School of Medicine, Shahid Bahonar Hospital, Kerman University of Medical Sciences, Kerman, Iran; 5 Department of Pediatric, School of Medicine, Afzalipour Hospital, Kerman University of Medical Sciences, Kerman, Iran; 6 Nursing Research Center, Kerman University of Medical Sciences, Kerman, Iran; 7 Department of Critical Care Nursing, Faculty of Nursing and Midwifery, Kerman University of Medical Sciences, Kerman, Iran

**Keywords:** Managerial approaches, Challenges, Covid-19, Pandemic, Qualitative study

## Abstract

**Background:**

During the covid-19 pandemic, hospitals have faced several challenges, so they need to identify and address effective management strategies to cope with these challenges that enhance their current knowledge to deal with similar challenges in the future. This study aimed to identify managerial strategies for dealing with Covid-19 pandemic challenges at a hospital in southeastern Iran.

**Methods:**

This qualitative content analysis study used purposive sampling to select eight managers, three nurses, and one worker from Shahid Bahonar Hospital. In this study, semi-structured interviews were used to collect data and Lundman and Graneheim’s approach was used to analyze them.

**Results:**

Three hundred fifty codes remained after constant comparison, compression, and merging. The results demonstrated one theme “Managerial reengineering in the healthcare system during the Covid-19 crisis”, two main categories, seven subcategories and, 19 sub- subcategories. The first main category was “The difficulty of managing challenges,“ including “Insufficient resources and physical space”, “Socio-organizational challenges” and, “Incompetence and unpreparedness of managers.“ The second main category was “Reforming the management duties.“ This category included “Planning and decision-making,“ “Organization,“ “Leadership and motivation,“ and “Monitoring and control.”

**Conclusions:**

Hospitals and managers were less prepared to cope with the Covid-19 crisis challenges due to health system organizations’ less attention to biological crises. Healthcare organizations can carefully evaluate these challenges, and the strategies managers adopt to deal with these problems. They also can identify the strategies’ strengths and weaknesses and propose more effective strategies. As a result, healthcare organizations will be better prepared for similar crises.

## Background

The Covid-19 pandemic was the most severe challenge, bringing one of the most critical conditions to healthcare organizations worldwide [1]. WHO declared the new Covid-19 outbreak a public health emergency in January 2020 and Covid-19 a global pandemic in March 2020 [2]. Despite global efforts to eradicate the Covid-19 pandemic, the disease spread rapidly, involving more than 210 countries in the first wave [3]. During this pandemic, healthcare organizations, including hospitals, were under tremendous pressure worldwide to limit the spread of Covid-19 [4]. The management of the Covid-19 crisis was very complicated and confusing for health policymakers and practitioners because of the unknown nature of the disease, its high contagiousness, and mortality of Covid-19 virus [5], and the lack of financial, material, and human resources [1]. An appropriate response to crises, especially emerging diseases, significantly impacted organizational readiness and success [5].

It is the responsibility of the hospital manager to cooperate, monitor, plan, determine the budget, and staff the hospital. Therefore, the awareness and performance of hospital managers will play an essential role in managing crises [6, 7]. Existing studies haven’t paid enough attention to the crisis management of epidemics and pandemics [8].

A qualitative study showed that nurses experienced inefficiency in the healthcare organization in supporting nurses, physical burnout, uncertainty, and psychological burden when caring for patients with Covid-19 [9]. One study addressed the ethical dilemmas and unexpected challenges of Covid-19 for nurses that could be affected by poor planning, preparation, organization, and leadership of some governments and health care systems [1]. Some studies demonstrated how governments controlled the disease, such as producing medicine and administrating vaccinations to reduce the spread of Covid-19 [10], identifying carriers and patients, closing markets, universities, and schools to disinfect them, training the public to practice good hygiene [11]. Most studies examined clinical issues [12] and therapeutic methods, the challenges of nurses and other health care workers [9], or the effects of Covid-19 on them [13, 14], as well as national and international measures [10, 11]. However, managerial challenges and approaches in hospitals can differ depending on the managers’ perspectives. One study found that innovation and creativity, a fast and agile organizational structure, and an ecosystem and network with open innovation prior to Covid-19 helped the organization better respond to the crisis [5]. As Iranian health managers and policymakers were unfamiliar with this large-scale epidemic, they controlled and managed it without prior planning [11]. More research is required to explore various aspects of this crisis and provide operational experiences to managers and policymakers. The coronavirus outbreak posed numerous challenges to healthcare organizations [5]. Managers must identify these challenges and implement effective measures to improve organizational capacities and standards, increase current knowledge and preparation, and use effective strategies in these crises. However, further studies can provide deep insight into these managerial challenges and strategies for dealing with this crisis and improving the current situation. Little information is available about the managerial challenges and approaches during the Covid-19 epidemic [9]; therefore, it is essential to understand the existing managerial challenges and solutions based on the managers’ experiences during the Covid-19 pandemic. Health policymakers and managers can overcome limitations and future challenges if they thoroughly understand these issues. This study aimed to identify managerial strategies for dealing with Covid-19 pandemic challenges at a hospital in southeastern Iran.

## Methods

### Study design and setting

This study aimed to investigate managerial challenges and strategies used to control Covid-19 in one of the most important hospitals in southeastern Iran. This qualitative descriptive-explorative study used a conventional content analysis [15] to obtain valid and reliable results from textual data, generate new knowledge and insight, present facts, and provide practical guidance [15]. The research setting was Shahid Bahonar hospital, the largest trauma, health, and treatment center in Karman, southeast Iran.

### Participants and sampling

This study included ten women and seven men aged 34–50 years. Managers (N = 8) had 10–20 years of work experience. We conducted five supplementary interviews with five managers again to complete some categories. Lastly, this study involved 12 participants and 17 interviews.

In order to examine managerial approaches, this study also used theoretical sampling to include three nurses and one worker employed in the Covid-19 ward, ICU Covid, and general wards. The sampling period was from December 2021 to July 2022. The educational supervisor provided information about the study and interviews to attract participants. Purposive sampling was used to select participants in this study, and sampling continued until data saturation, which meant that new data were unavailable [16]. We reached saturation after ten interviews, but we conducted two more interviews to ensure the data collection had not added additional data to the study. We selected the participants based on the maximum variation in age, gender, and working experience. As criteria for inclusion, those working in the hospital during Covid-19, managers handling Covid-19 challenges, and those with good physical and mental health and mastery of Persian were considered. The participants were already aware of the managerial challenges and solutions to the Covid-19 crisis. However, the researcher clarified these concepts before the interview with the participants.

### Data collection procedure

The study collected data through semi-structured and face-to-face interviews with open-ended questions. The second author conducted interviews at a convenient time and location, and the third author supervised the interview process. Each interview lasted approximately 35 to 45 min. The researcher encouraged the participants to share their experiences. The researcher recorded and transcribed all the interviews. Table 1 shows an example of an interview question.

**Table 1:** A sample interview question

number	questions
1	“What challenges did you face during the Covid-19 crisis? ”
2	“Could you please elaborate on these challenges?
3	“How did you handle the Covid-19 challenges? ”
4	What other strategies have you adopted to overcome challenges?”
5	Could you please describe the strategies used in planning and decision-making?
6	Could you please describe the strategies used in the area of organization?
7	Could you please describe the strategies used in leadership and motivation?
8	Could you please describe the strategies used in monitoring and control?

### Data analysis

The researcher collected and analyzed data simultaneously from December 2021 to August 2022. The data included interviews with 12 participants (managers, nurses, and one worker) about their managerial challenges and approaches to overcoming the Covid-19 pandemic. The second researcher transcribed all interviews verbatim. This study used Graneheim and Lundman’s approach to analyze data [15]. The researcher focused on the hidden content of data and meanings when interpreting the text [15]. We read the interviews several times, identified and condensed meaning units, and coded and categorized them into subcategories and categories to find a theme that describes the meaning of the data. We used MAXQDA version 10 to manage the data. MD and ZE analyzed the interviews. The authors checked and approved all categories and themes. ZE, ZN, MJ, MAN, MAP, PM, and MD contributed to the final written report’s composition, review, and correction. Table 2 illustrates an example of the analysis process.

**Table 2:** An example of qualitative content analysis process

main category	Subcategory	sub-subcategories	codes	Meaning unit
The difficulty of managing challenges	Insufficient resources and physical space	Budget and equipment shortages	Inadequacy of personal protective equipment	“We faced a severe shortage of personal protective equipment in the early months. During each shift, they provided only one mask and gown; if it became contaminated, we had to use it until the end.” (Participant No. 3).
		Deficiency of skilled human resources	Nursing staff shortage	“We were facing a staff shortage as a result of the increased number of beds. These shortages have always existed, but they became more visible and problematic during this crisis.” (Participant No.12)
		Inadequate and unfavorable physical conditions	Poor equipment quality	“Our masks and disinfectants were of poor quality and caused skin irritation”. (Participant No. 11).

### Trustworthiness

The current study used Guba and Lincoln’s criteria to determine data trustworthiness [15]. The researcher’s long engagement with the data, maximum variation sampling, member check, and peer check all contribute to credibility. Some participants read a summary of the analyzed data to compare it to their own experiences and attitudes. External checks confirmed data on the texts of interviews, codes, and extracted categories. Data transferability was confirmed by describing the data collection method, data analysis process, and direct quotations.

## Findings

The results showed one theme “Managerial reengineering in the healthcare system during the COVID-19 crisis”, two main categories, seven subcategories and, 19 sub-subcategories (Table 3). The main categories were “The difficulty of managing challenges”, and “Reforming the management duties”. Three hundred fifty codes remained after constant comparison, condensation and merging.

**Table 3:** Main category, categories, and subcategories of challenges and management approaches during the covid-19 pandemic

theme	main categories	Subcategories	sub-subcategories
Managerial reengineering in the healthcare system during the Covid-19 crisis	The difficulty of managing challenges	Insufficient resources and physical space	Budget and equipment shortages
			Deficiency of skilled human resources
			Inadequate and unfavorable physical conditions
		Socio-organizational challenges	An unprecedented increase in staff workload
			Unprecedented death tension
			Top managers’ unreasonable expectations
			The resistance to inevitable change
		Incompetence and unpreparedness of managers	A lack of knowledge and Poor performance
			Resource mismanagement
	reforming the management duties	Planning and decision-making	Development of counseling and educational programs
			Redefining executive and care duties
			Enhancing the response to subsequent crises
			Compensating for resource shortages
		Organization	Reducing workload and organizing resources
			Facilities and physical space organization
		Leadership and motivation	Interactive management
			Culture of teamwork promotion
		Monitoring and control	Monitoring resource distribution and consumption
			Measures for isolation and prevention

## Theme: managerial reengineering in the healthcare system during the Covid-19 crisis

### Category 1: The difficulty of managing challenges

It was unexpected that the deadly spread of the Covid-19 pandemic outstripped the hospital’s available resources and left healthcare managers in shock. In addition to inadequate preparation, managers were under unprecedented pressure regarding providing a wide array of resources and equipment and organizational and public pressures. Due to inefficient approaches, they were challenged to manage this epidemic beyond their capabilities. This category includes the subcategories of “Insufficient resources and physical space,” “Socio-organizational challenges,” and “Incompetence and unpreparedness of managers.”

#### Subcategory A: insufficient resources and physical space

Most participants admitted that in previous crises, such as the simultaneous admission of many trauma patients, they managed this crisis well by increasing the number of beds and bringing in additional helpers. Managers could provide sufficient space, equipment, and power in such critical conditions. However, the duration of these crises was short, and the patient’s assignments and conditions were determined quickly. However, during the Covid-19 crisis, they faced a severe lack of material, human and physical resources. Below, we have outlined these challenges in three sub-subcategories.

##### Sub-subcategory 1. Budget and equipment shortages

Most participants acknowledged the lack of equipment to establish new departments during the Covid-19 outbreak. Additionally, there was a severe shortage of personal protective equipment at the beginning of the outbreak. Due to the sanctions and the economic situation in the country, they did not have sufficient funds to provide the necessary equipment.

“We faced a severe shortage of personal protective equipment in the early months. During each shift, they provided only one mask and gown; if it became contaminated, we had to use it until the end.” (Participant No. 3).

“We could not purchase medicine and equipment due to a lack of funds”. (Participant No. 2).

##### Sub-subcategory 2. Deficiency of skilled human resources

Further, participants acknowledged that during the Covid-19 crisis, the lack of human resources multiplied for health system organizations. There are fewer applicants for hospital jobs due to the disease’s unknown and fatal nature. Consequently, there was a severe shortage of nurses and specialists. As a result, managers used non-nurse personnel, including anesthesiologists and operating room staff, which posed other challenges. Regarding the lack of workforce, the participants acknowledged the following:

“We were facing a staff shortage as a result of the increased number of beds. These shortages have always existed, but they became more visible and problematic during this crisis.” (Participant No.12).

“We were severely understaffed and worked 240 hours per month. Staff with Covid-19 suffered from physical weakness, respiratory problems, and walking disorders. After sick leave, they had to return to work with the same workload and complications.” (Participant No. 10).

##### Sub-subcategory 3. Inadequate and unfavorable physical conditions

During this unprecedented crisis, managers have struggled to provide a safe environment for their employees and patients. The Covid-19 center was full, so patients were referred to other hospitals, including this one. Moreover, the hospital’s space was inadequate for new wards and ICUs. Regarding the inadequate and unfavorable physical conditions, the participants acknowledged the following:

“There was a severe shortage of ICU beds. There wasn’t enough space. Our choice of space was far from the operating room. ICU setting was not standard. The ventilation was poor.” (Participant No. 1).

“Our masks and disinfectants were of poor quality and caused skin irritation”. (Participant No. 11).

#### Subcategory B: socio-organizational challenges

Most participants acknowledged staff feared working on Covid-19 wards. Employees protested against the closure and merger of the wards and their transfer to Covid-19. In addition, other patients were dissatisfied with the closure of elective surgery wards and the admission of Covid-19 patients to the trauma center. Patients’ high death rate had led to aggressive reactions from their companions and tension among staff and doctors. The relatives of patients were unaware of the restrictions regarding visits, so they were reluctant to comply with them. Clinical training was halted due to the increased number of Covid-19 patients, and managers faced protests from medical students. Also, higher-level managers expected the hospital to establish a Covid-19 ward within a short time, even though the hospital specialized primarily in surgical and trauma care and was under-equipped for infectious disease care. Below, we have outlined these challenges in four sub-subcategories.

##### Sub-subcategory 1. An unprecedented increase in staff workload

Participants stated that the simultaneous admission of large Covid-19 and traumatized patients doubled the staff workload. The Covid-19 patients were also in feeble health. As a result, the staff had to deal with complex medical care, making their work harder and increasing their workload.

“There was no decrease in trauma patients despite the Covid-19 crisis. Trauma patients were also admitted along with Covid-19 patients. Due to this, our workload was doubled. It makes preventing disease spread and isolation difficult.” (Participant No. 7).

##### Sub-subcategory 2. Unprecedented death tension

According to most participants, they witnessed unprecedented deaths of patients of all ages. Treatment efforts were unsuccessful. Medical staff were not able to prevent the unexpected death of patients. Every day, the number of deaths increased. As a result, companions displayed aggressive behavior and tension among the medical staff increased.

“In the outbreak of the alpha strain of the Covid-19 virus, there were no effective treatments. As a result, many patients died in ICUs.” (Participant No. 5).

##### Sub-subcategory 3. Top managers’ unreasonable expectations

Most participants stated that other hospital heads did not understand the different missions of the trauma center. The high-level managers expected them to establish the Covid-19 Ward within hours. High-level managers forced them to provide the workforce to other Covid-19 centers despite a lack of workforce.

“Managers stated that it would be easy to establish wards and that we would have to establish another ward until evening. However, it was difficult. We required beds, mattresses, a workforce, devices, and an oxygen supply.” (Participant No. 6).

##### Sub-subcategory 4. The resistance to inevitable change

Most participants stated that personnel resisted working in Covid-19 Wards. Medical students protested managers’ decision to halt clinical training due to many Covid-19 patients. There was a strong protest by the relatives of trauma patients, who displayed aggressive behavior in fear of the infection of their loved ones. Despite the warnings, officials and the general public had not yet recognized the seriousness of the disease. The relatives of patients were unaware of the restrictions regarding visits, so they were reluctant to comply with them.

“Staff were under stress because personal protective equipment effectiveness was still unknown. Employees did not want to work in the Covid-19 Wards because they were afraid. As a result, we met many challenges.” (Participant No. 2).

“Due to the shortened clinical training courses and virtual classes, students protested.” (Participant No. 7).

#### Subcategory C: incompetence and unpreparedness of managers

Most participants said most managers did not sufficiently understand biological crisis management. This unknown pandemic left hospital managers unprepared. Furthermore, their previous strategies failed to manage challenges. In the early stages of the Covid-19 pandemic, managers could not perform well and took action too late. These factors led to their failure to resolve the crisis. Below, we have outlined these challenges in two sub-subcategories.

##### Sub-subcategory 1. A lack of knowledge and poor performance

Several participants acknowledged that a system’s tendency to surprise indicates its vulnerability. Managers lacked crisis management knowledge. Due to the new and unknown nature of this pandemic, this manager’s weakness was accentuated.

“As Covid-19 peaked, some managers were bewildered because of their lack of crisis management competence, and they worsened the situation by taking mistaken measures.” (Participant No. 3).

“Some challenges were exacerbated by some managers’ failure to act on time at the beginning of Covid-19.” (Participant No. 9).

##### Sub-subcategory 2. Resource mismanagement

According to most participants, equipment distribution between hospitals and wards was improper, resulting in severe shortages of medicine and equipment in some hospitals and wards. Most recruited workers were sent to the Covid-19 Center’s particular hospital, while other hospitals suffered from staff shortages.

“There was an inefficient distribution of resources. The majority of the equipment was sent to Covid-19 Center Hospital. At the same time, we suffered from a severe shortage of ventilators and medicines.” (Participant No. 4).

### Category 2: reforming the management duties

According to the analysis of the findings, managers adopted various strategies in response to the challenges posed by Covid-19. These strategies were categorized based on the managers’ four duties: planning and decision-making, organizing, leading and motivating, and monitoring and controlling, which will be discussed in more detail below.

#### Subcategory A: planning and decision-making

Most participants admitted that they developed a series of educational and consulting programs to improve the performance of managers and less experienced employees. Additionally, they increased public awareness through educational advertising and developed educational programs for patients and their companions. A care and executive guidelines and facilitating protocol were developed to reduce deaths, improve decision-making speed, and improve care quality. Managers’ and employees’ duties were formulated according to the crisis conditions, and the division of work was announced. Below, we have outlined these strategies in four sub-subcategories.

##### Sub-subcategory 1. Development of counseling and educational programs

Several participants reported that they were able to overcome some challenges by predicting and developing educational and advisory content. Some of these challenges were the lack of expert staff, inadequate managerial knowledge and performance, and insufficient public awareness. As a result, disease mortality rates were also reduced. Specifically, they analyzed the needs of personnel and managers, prepared training and consulting course content, developed a schedule for implementing programs, developed educational programs to inform individuals, trained patients upon discharge to prevent frequent visits, and utilized virtual networks and media to convey educational information.

“We trained both newly hired and non-nursing personnel on how to work with ventilators, non-invasive mechanical ventilation, and do triage, and then they started working in the Covid-19 Wards and emergency departments.” (Participant No. 8).

“Broadcasting organizations were very effective in teaching masks and hand washing.” (Participant No. 7).

##### Sub-subcategory 2. Redefining executive and care duties

Most participants reported that they provided job descriptions and divisions of work, identified upcoming care needs, notified personnel of the updated national orders for Covid-19 immediately and developed revised guidelines. As a result of their predictions and implementation of these measures, the hospital has been able to enhance staff and managers’ knowledge and performance and be more efficient with limited resources. Consequently, care quality improved, and disease mortality rates decreased.

“The national oxygen therapy guideline was revised and sent to all wards to prevent oxygen waste.” (Participant No. 2).

##### Sub-subcategory 3. Enhancing the response to subsequent crises

In most cases, participants reported that they analyzed previous Covid-19 peaks to identify unsolved problems. By providing appropriate solutions to challenging problems, they prepared managers to manage following Covid-19 peaks more effectively and overcome their incompetence and lack of preparedness. It resulted in improved care quality and a decrease in disease mortality.

“We added a 450-liter container to the oxygen generators before the second peak because we anticipated a more severe peak and the need for more oxygen supply.“ (Participant No.2).

“The Delta virus caused the second peak. The experiences we gained were very beneficial. We faced many deaths in Covid’s ICU, but we reduced them by two solutions in the next peak.“ (Participant No.1).

##### Sub-subcategory 4. Compensating for resource shortages

Several participants reported that they had prepared a new budget plan, conducted meetings with the ministry, and advertised for charities to raise funds. Taking these actions was necessary to overcome the challenges created by budget shortages. Thus, shortages of medicine and equipment could be alleviated.

“After the Ministry’s correspondence with the pharmaceutical company, we could purchase our medicine because we did not have enough funds.” (Participant No.5).

#### Subcategory B: organization

According to most managers, they organize various resources efficiently at this field. As well as categorize activities according to the provided job descriptions and divisions of responsibilities. Taking these actions was necessary to overcome the challenges created by a shortage of skilled human resources, an unprecedented increase in workload, equipment shortages, and unfavorable working conditions. Below, we have outlined these strategies in two sub-subcategories.

##### Sub-subcategory 1. Reducing workload and organizing resources

Several participants reported that they had implemented some measures to cope with the lack of skilled human resources, an unprecedented increase in staff workload and equipment shortages challenges. They adjusted and distributed the workers within the wards in an appropriately balanced manner, sort activities according to job descriptions and responsibilities, reduced in the number of beds on other clinical wards, reduced patients’ re-visits by providing virtual medical services, closed of the elective wards, establishing an ambulatory and home care facility, distributed experienced personnel throughout the wards, and preventing severe conditions by early diagnosis and timely action, developed a facilitator protocol, distributed consumables according to needs, and recruited volunteers. This strategy also reduced disease mortality and improved care quality.

“In addition to routine triage in the emergency department, we started a respiratory triage to evaluate Covid-19 patients.” (Participant No. 6).

“For example, the workforce was adjusted in accordance with the severity and low workload of the wards, and we used people who wanted to volunteer.” (Participant No. 5).

##### Sub-subcategory 2. Facilities and physical space organization

Several participants reported that they had implemented some measures to cope with inadequate and unfavorable physical conditions challenges. They established new wards, enhanced the physical environment of Covid-19 Wards and ICUs.

“To admit Covid-19 patients, we opened three wards with 70 active beds. In addition, eight ICU beds were made available.” (Participant No.1).

#### Subcategory C: leadership and motivation

Most participants indicated they reduced employee resistance to inevitable changes, reduce employee tension, increase motivation via interactive management and promote a teamwork culture. Thus, personnel always strive to complete their tasks in the best possible manner and to the highest standard. Below, we have outlined these strategies in two sub-subcategories.

##### Sub-subcategory 1. Interactive management

Many participants reported that they had implemented measures to reduce resistance to inevitable changes, decrease employee tension, and increase motivation. In order to do so, they adopted a dialogue strategy, resolved conflicts openly, and continuously encouraged and motivated their employees.

“My first step was to gather the disgruntled staff and explain the difficult conditions to them, as well as provide incentives for them.” (Participant No.10).

##### Sub-subcategory 2. Culture of teamwork promotion

Several participants reported that they had implemented measures to decrease resistance to inevitable change, decrease staff tension, and create order and coordination. As a result, healthcare quality and disease mortality improved. In order to do so, they created friendly cooperation between doctors and nurses, provided support by experienced personnel to less experienced personnel and a team visit by a variety of specialists.

“Doctors and nurses were invited to work together. Many patients did not require mechanical ventilation thanks to the efforts of the anesthesiologists, infectious disease specialists, and other experienced staff who worked together to keep them from getting more complications.” (Participant No. 1).

#### Subcategory D: monitoring and control

Several managers admitted that they evaluated optimal resource distributions and consumptions through control and monitoring to counter equipment shortages and inexperienced staff challenges. Managers could identify and correct deviations and weaknesses by periodically reviewing their employees’ performance and tools. Managers also implemented measures to prevent disease and illness, reducing sick leave. They reduced staff workload by preventing the spread of disease. These measures also reduced disease mortality and improved care quality. Below, we have outlined these strategies in two sub-subcategories.

##### Sub-subcategory 1. Monitoring resource distribution and consumption

In most cases, participants reported implementing measures to counter equipment shortages, medication shortages, and inexperienced human resources challenges. It is their responsibility to ensure the proper distribution of consumables and medicines. They examine equipment performance regularly, monitor oxygen consumption and rare drugs, and form teams to assess personnel performance.

“During the pandemic, personnel practices were evaluated, and unskilled ones were trained and supervised by skilled personnel.” (Participant No.4).

##### Sub-subcategory 2. Measures for isolation and prevention

The participants reported that they had implemented measures to prevent the spread of disease, illness, and sick leave among employees and to manage the shortage of human resources. Covid-19 Wards were separated from other clinical wards, patients were screened and quarantined, all visits by patients’ companions and others were restricted, employees were vaccinated, and a system for reporting positive cases was established.

“With the assistance of the information technology department, we programmed an alarm into the HIS system to indicate that the patient was suspicious or infected. When a nurse on duty saw a suspected patient’s name in the system, they followed the protocols more closely.“ (Participant No. 9).

## Discussion

This study aimed to examine managerial challenges and approaches in one of the most important hospitals in southeastern Iran during the Covid-19 epidemic. Managers faced many challenges and used various approaches to address them. Participants’ experiences suggested the main theme of “Managerial reengineering in the healthcare system during the Covid-19 crisis” with two main categories of “The difficulty of managing challenges” and “reforming the management duties.”

### The difficulty of managing challenges

The study results showed that managers were unprepared, with a severe lack of resources and equipment and ineffective approaches during the pandemic. A study also showed that many countries faced several challenges, such as insufficient resources, insufficient personal protective equipment, a high number of referrals, lack of human resources, and unpreparedness of the health system to deal with this pandemic [9]. Covid-19 is highly contagious, and its transmission was unknown at first, so many unprotected or inadequately protected healthcare workers became infected during the outbreak of Covid-19 [17]. Transmission is possible during the incubation period, which makes it difficult to control its spread [18]. They caused managers to have difficulty controlling this crisis. Managers’ options became more limited because of organizational and public pressures. Evidence suggests that trust is essential during infectious disease outbreaks because it can influence perception and interventions, such as physical distancing and information-seeking behavior [19]. Several aspects of the Covid-19 pandemic, including the existing knowledge gap about the cause, modes of transmission, treatment, and high mortality of Covid-19 and its unknown nature, posed challenges to building trust. One study showed that a creative organizational structure before the outbreak of Covid-19 helped managers become prepared and respond to this crisis better [5]. The study hospital provided trauma services to patients without prior experience with biological crises. As a result, the previous organizational structure was insufficient to manage this crisis easily. In addition, managers’ inexperience with biological pandemics and their inability to make decisions in crisis, improper management of resources, underestimation and delay in decision-making, and previous inefficient approaches made it difficult for the organization and managers to manage this crisis and provide a better response. Knowledge management is a strategic investment in healthcare organizations. More challenges and failures were available in the management of the Covid-19 crisis due to inefficient approaches, so creative and innovative solutions were necessary to obtain successful experiences.

### Reforming the management duties

The study results showed that managers adopted various strategies in response to the challenges posed by Covid-19. By developing educational and consulting programs, increasing public awareness, developing guidelines and protocols, and re-forming the role of managers and employees, managers were able to address some challenges in the planning and decision-making field. One study also found that personnel training was effective measures to solve the challenges of the Covid-19 crisis [20]. In this regard, another study to overcome the challenges of Covid-19 in the health system, which recommends assessing learners’ needs in educational programs, emphasizes the importance of balance and flexibility to meet the multiple demands of educational programs, institutions, and the general public [21]. Another study addressed the role of training employees and doctors in managing financial and human resources [22]. Another study showed that using an algorithm to predict the need for ventilation in Covid-19 patients helped identify patients in need of invasive ventilation and improve the outcomes of Covid-19 patients [23]. A study also mentioned using trained risk analysis experts in strategic decisions, regular data collection and analysis, and specialized resources, including universities, to create and improve essential capacities and respond to the crisis [19].

Managers also organize various resources efficiently and categorize activities according to overcome the challenges created by a shortage of skilled human resources, an unprecedented increase in workload, equipment shortages, and unfavorable working condition. In this regard, a study recommended developing a comprehensive human resources management plan for COVID-19 and personnel support packages to address the challenges associated with COVID-19 [24]. Covid-19 was successfully combated in a study that employed appropriate strategies, including transparent organizational structures to respond to critical threats and coordinate activities, key personnel roles, quick responses, and more evidence to transform threat assessment data into a standard and strategic operational approach [19]. Managing financial and human resources also played a crucial role in successfully overcoming the challenges of Covid-19 [22]. Another study focused on organizational relationships, which resulted in coordinated activities, rapid information sharing, and friendly communication with the mass media, which delivered accurate information during the Covid-19 pandemic [19]. Also, a study found that implementing appropriate human resource management strategies increases mental well-being, satisfaction, productivity, motivation, and safety in the workplace during the Covid-19 crisis [25].

In addition, managers reported that they could reduce resistance to inevitable changes, decrease employee tension, and increase motivation by adopting a dialogue strategy, resolving conflicts openly, and continuously encouraging and motivating their employees. One study introduced strategies such as negotiation sessions and feedback meetings to reduce Covid-19’s psychological impact and overcome employees’ psychological distress [26]. According to another study that examined the importance of collaboration during the Covid-19 pandemic, a dynamic workflow requires clear communication and adherence to policies and procedures [27].

In addition, managers evaluated the optimal distribution and consumption to identified deviations and weaknesses and corrected them. Also implemented measures to prevent disease and illness, reducing sick leave. In this regard, a study in Taiwan showed that hospitals used strategies to prevent the spread of Covid-19, including quick identification of suspicious cases, temporary preventive or control measures, installation of infrared cameras at hospital and emergency department entrances to identify people with fever, limited visits, and closure of all hospital entrances except those for personnel [22]. Another study also found that personal protective equipment, N95 masks, disinfection and hand hygiene, and a sufficient supply of resources were effective measures to solve the challenges of the Covid-19 crisis [20].

According to a comparison of hospital managers’ experiences with those conducted in various countries, these studies did not examine the management strategies for coping with COVID-19 challenges from the perspective of management tasks such as planning, decision-making, organization, leadership, or motivation. Most studies used strategies such as human resource management, limiting visitors, establishing new departments, managing beds, and training personnel. These studies did not use some of the strategies used in this study, such as training volunteers for non-medical purposes or obtaining financial support from benefactors. Policymakers and managers should consider some alternative strategies. For example, in this hospital, risk analysis experts with training in strategic planning should be considered.

### Limitations and strengths of the study

The small sample size and data collection from only one center are the limitations of this study. Furthermore, since the study was conducted in just one hospital, its generalizability is limited. One of the strengths of this study is the identification of managerial challenges and approaches, which can help manage the challenges of biological epidemics in other countries and contexts.

## Conclusion

During the Covid-19 pandemic, management challenges were identified, such as insufficient resources and physical space, socio-organizational challenges, and managerial incompetence and unpreparedness. As a result of Covid-19, managers adopted a variety of strategies. Based on the four responsibilities of managers, these strategies were categorized into planning and decision-making, organizing, leading and motivating, and monitoring and controlling. While the issues discussed in this study may apply to other countries and contexts as well, it is recommended that more interdisciplinary and multinational research be conducted on prevention and preparedness strategies before the outbreak of the Covid-19 pandemic as well as other successful strategies for managing the crisis.

## Data Availability

The datasets used for the current study are available from the corresponding author upon request.
